# Association of cancer metabolism-related proteins with oral carcinogenesis – indications for chemoprevention and metabolic sensitizing of oral squamous cell carcinoma?

**DOI:** 10.1186/1479-5876-12-208

**Published:** 2014-07-21

**Authors:** Martin Grimm, Marcel Cetindis, Max Lehmann, Thorsten Biegner, Adelheid Munz, Peter Teriete, Wiebke Kraut, Siegmar Reinert

**Affiliations:** 1 Department of Oral and Maxillofacial Surgery, University Hospital Tuebingen, Osianderstrasse 2-8, Tuebingen 72076, Germany; 2 Department of Pathology, University Hospital Tuebingen, Liebermeisterstrasse 8, Tuebingen 72076, Germany; 3 Cancer Research Center, Sanford-Burnham Medical Research Institute, 10901 North Torrey Pines Road, La Jolla, CA 92037, USA

**Keywords:** Oral squamous cell carcinoma, Tumor metabolism, Glycolysis-related proteins, Mitochondrial oxidative phosphorylation, Carbohydrate-restricted diets, Targeted anti-mitochondrial therapy

## Abstract

**Background:**

Tumor metabolism is a crucial factor for the carcinogenesis of oral squamous cell carcinoma (OSCC).

**Methods:**

Expression of IGF-R1, glycolysis-related proteins (GLUT-1, HK 2, PFK-1, LDHA, TKTL1), mitochondrial enzymes (SDHA, SDHB, ATP synthase) were analyzed in normal oral mucosa (n = 5), oral precursor lesions (simple hyperplasia, n = 11; squamous intraepithelial neoplasia, SIN I-III, n = 35), and OSCC specimen (n = 42) by immunohistochemistry and real-time polymerase chain reaction (qPCR) analysis in OSCC cell lines. Metabolism-related proteins were correlated with proliferation activity (Ki-67) and apoptotic properties (TUNEL assay) in OSCC. Specificity of antibodies was confirmed by western blotting in cancer cell lines.

**Results:**

Expression of IGF-R1, glycolysis-related proteins (GLUT-1, HK 2, LDHA, TKTL1), and mitochondrial enzymes (SDHA, SDHB, ATP synthase) were significantly increased in the carcinogenesis of OSCC. Metabolic active regions of OSCC were strongly correlated with proliferating cancer (Ki-67+) cells without detection of apoptosis (TUNEL assay).

**Conclusions:**

This study provides the first evidence of the expression of IGF-R1, glycolysis-related proteins GLUT-1, HK 2, PFK-1, LDHA, and TKTL1, as well as mitochondrial enzymes SDHA, SDHB, and ATP synthase in the multi-step carcinogenesis of OSCC. Both, hypoxia-related glucose metabolism and mitochondrial oxidative phosphorylation characteristics are associated with the carcinogenesis of OSCC. Acidosis and OXPHOS may drive a metabolic shift towards the pentose phosphate pathway (PPP). Therefore, inhibition of the PPP, glycolysis, and targeted anti-mitochondrial therapies (ROS generation) by natural compounds or synthetic vitamin derivatives may act as sensitizer for apoptosis in cancer cells mediated by adjuvant therapies in OSCC.

## Introduction

Cancer is regarded as an acquired genetic disease. The genetic model of multistep carcinogenesis describes the rise of malignant tumors from a single transformed cell (monoclonal theory of carcinogenesis) and subsequent development through morphologically and clinically detectable precancerous stages [1]. The carcinogenesis of oral squamous cell carcinoma (OSCC) is a highly complex multifocal process that occurs when squamous epithelium is affected by several genetic alterations [2]. Understanding the mechanistic basis await the availability of molecular tools to experimentally and selectively manipulate this multistep process with subsequent clinical implications for therapy of precursor lesions and OSCC.

OSCC is an aggressive tumor with low response to chemotherapy and basic resistance to most standard of care anticancer drugs [3,4]. Tumor metabolism [5] with a special focus on increased hypoxia/glycolytic activity is regarded as a crucial factor for the carcinogenesis of OSCC and is associated with radio- and, chemotherapy resistance, as well as tumor recurrence [6-9].

Cancer can be considered as integrated metabolic ecosystem and includes several pathways of carcinogenesis associated with metabolic phases of transformation [10]. Glycolysis [11], mitochondrial oxidative phosphorylation (OXPHOS) [12], and glutaminolysis have been shown to play key roles in tumor metabolism. Mitochondria have an important role in carcinogenesis due to their roles in mediating apoptosis [13]. They act as a major source of endogenous reactive oxygen species (ROS) that escape from the electron transport chain (ETC.) during OXPHOS [14]. Although glycolysis is a major characteristic of tumor cell metabolism this pathway alone cannot account for energy usage in all types of cancer cells. Finally, the dominant metabolic process can be either glycolysis or mitochondrial oxidative metabolism based on the tumor type [15]. Both metabolic phenotypes have been associated with subsequent nutritional consequences [16-19].

The generation of adenosine triphosphate (ATP) in glycolysis has a lower efficiency, but a faster rate than OXPHOS [11,20]. This enhanced rate of ATP generation has been postulated to be beneficial for rapidly proliferating cells. However, several studies have suggested that OXPHOS is the major source of cellular ATP in proliferating and non-proliferating [21] cancer cells [11,21-23].

A recent study by Vander Heiden [24] indicated that the induction of the Warburg effect in cancer cells is more the consequence of the activation of protooncogenes (*e.g.*, Myc), transcription factors (*e.g.*, hypoxia-inducible factor-1, HIF-1), and signaling pathways (*e.g.*, PI3K), as well as the inactivation of tumor suppressors (*e.g.* p53) rather than the primary generation of much needed energy [11]. Moreover, it has been stated that tumor cells profit from the enhanced glycolytic activity in glycolytic intermediates, which are shunted into subsidiary pathways (*e.g.* by the pentose phosphate pathway [PPP]) to fuel metabolic pathways that generate *de novo* nucleotides, lipids, amino acids, and nicotinamide adenine dinucleotide phosphate (NADPH) [11,25,26]. Frezza *et al.*[12] showed that defects in mitochondrial enzymes or complexes within the electron transport chain are not frequently observed in cancer. Therefore, investigation of OXPHOS provides a clear rational for future anti-cancer therapy strategies in OSCC [27].

Today, it is estimated that more than 30% of all tumor entities may be due to dietary factors [17]. Studies have clearly linked diabetes and obesity to cancer [28]. Hyperinsulinemia leads to increased production of insulin-like growth factor-1 (IGF-1) [29], which activates insulin-like growth factor-1 receptor (IGF-1R). IGF-1R is a receptor tyrosine kinase (RTK) that stimulates protein synthesis by activating the mammalian target of rapamycin (mTOR), and in turn mTOR mediated upregulation of glycolytic enzymes may promote tumor development [30,31]. Therefore, the IGF-1R pathway is an emerging therapeutic target in oncology [32-34] but has not yet been described for the carcinogenesis of OSCC.

Hexokinase 2 (HK 2) is expressed in insulin-sensitive tissues such as muscle and adipose [11], is one of the rate-limiting enzymes of glucose catabolism in tumor cells, is upregulated in many cancers [35,36], and was recently described for OSCC [37]. Phosphofructokinase-1 (PFK-1) [38] is a key enzyme in glycolysis where it forms a rate-limiting step, but its expression has not been described for OSCC. Among glycolytic enzymes PFK-1 has been more extensively studied than other enzymes, which is likely to be due to its various regulatory mechanisms.

Recently, we have demonstrated glucose transporter 1 (GLUT-1) (solute carrier family 2 [facilitated glucose transporter], member 1 [SLC2A1]) [9], transketolase-like-1 (TKTL1) [7], and lactate dehydrogenase A (LDHA/LDH5) [39] as adverse prognostic factors for the survival of patients with OSCC. However, the expression of GLUT-1, HK 2, PFK-1, LDHA, and TKTL1 during a multi-step carcinogenesis has not been described yet.

More recently, characterization of OXPHOS in cancer was performed by describing succinate dehydrogenase SDHA, SDHB (respiratory complex II in mitochondria), and ATP synthase (respiratory complex V in mitochondria) [40,41]. None of these enzymes have yet been described for OSCC.

The purpose of this study was to examine the relationship between metabolism-related proteins [8] with a multistep carcinogenesis. This is the first study describing glycolysis-related PFK-1, OXPHOS-related SDHA, SDHB, and ATP synthase in OSCC.

## Materials and methods

### Patients and tumor specimen

The records of healthy individuals (normal oral mucosal tissues, n = 5), patients with oral precursor lesions (simple hyperplasia, n = 11; squamous intraepithelial neoplasia SIN I, n = 5; SIN II, n = 9; SIN III, severe dysplasia, n = 10; SIN III, carcinoma *in situ*, n = 11), and patients with invasive OSCC (n = 42) were retrospectively assessed from January 2009 to December 2013. The diagnosis of normal oral mucosal tissues, precursor lesions, and invasive squamous cell carcinoma was confirmed by the department of Pathology, University Hospital Tuebingen. The material was archival formalin-fixed, paraffin-embedded tissue from routine histopathological work-ups. The material has been stored with permission of the local ethics committee of the University Hospital Tuebingen (approval number: 562-2013BO2), after informed consent obtained from the patients prior to surgical resection. Tumor blocks of paraffin-embedded tissue were selected by experienced pathologists, evaluating the routine H&E stained sections. Sections from all available tissues underwent histopathological assessment, blinded to the prior histopathology report. Serial tissue sections (2 μm thickness) were cut from formalin-fixed paraffin-embedded (FFPE) blocks on a microtome and mounted from warm water onto adhesive microscope slides. First, we assessed H&E sections (Additional file 1: Figure S1) from each tissue section to differentiate between normal tissue, precursor lesions, tumor cell areas, stromal areas, and infiltrating immune cells. Oral precursor lesions were classified according to WHO criteria [1]. Tumor staging was performed according to the 7th edition of the TNM staging system by the UICC/AJCC of 2010 [42]. Grading of OSCC was defined according to WHO criteria [43].

### Staining procedure and quantification of immunohistochemistry

The antibodies used for immunohistochemistry are shown in Additional file 2: Table S1. We stained for IGF-R1β, glycolysis-related proteins GLUT-1, HK 2, PFK-1, LDHA, TKTL1, mitochondrial enzymes SDHA, SDHB, ATP synthase, and proliferation characteristics Ki-67 in serial sections (Additional file 2: Table S1). Staining was performed on serial sections of 2 μm thickness as previously described [39].

Five representative high power fields (1 HPF = 0.237 mm^2^, original magnification: x200-fold) were analyzed for IGF-R1β, GLUT-1, HK 2, PFK-1, LDHA, TKTL1, SDHA, SDHB, and ATP synthase expression in normal tissue, oral precursor lesions, tumor tissue and averaged, respectively. The extent of the staining, defined as the percentage of positive staining areas of tumor cells in relation to the whole tissue area, was semi-quantitatively scored on a scale of 0 to 3 as the following: 0, <10%; 1, 10–30%; 2, 30–60%; 3, >60%. The intensities of the signals were scored as 1+ (weak), 2+ (intermediate), and 3+ (strong). Then, a combined score (0–9) for each specimen was calculated by multiplying the values of these two categories [44]. Cases were classified as negative, 0 points, positive, 1–9 points. Two observers blinded to the diagnosis performed scoring on identical sections marked by circling with a water-resistant pencil and finally with diamond-tipped pencil on the opposite side of the microscopic slide. Pictures were analyzed using a Canon camera (Krefeld, Germany). The photographed images were imported into the Microsoft Office Picture Manager.

### *In situ* detection of apoptosis

Apoptotic cells and bodies were detected by the terminaldeoxynucleotidyl transferase-mediated deoxyuridinetriphosphate nick-end labeling (TUNEL) method (ApopTag^®^ Plus Peroxidase *In Situ* Apoptosis Kit, Chemicon, Planegg-Muenchen, Germany). The TUNEL assay is regarded as the ‘gold standard’ in apoptosis detection and was performed as described previously [45-47].

### Cell culture, western blot and densitometric quantification

BICR3 and BICR56 OSCC cell lines [9,48] were cultured in DMEM F-12 medium (Invitrogen, Belgium) containing 10% fetal calf serum (Sigma-Aldrich, Germany), 1% fungicide, and penicillin/streptomycin (Biochrom, Germany) at 37°C and 5% CO_2_.

IGF-R1β, HK 2, PFK-1, LDHA, SDHA, and SDHB antibody specificity was confirmed by western blot analysis in BICR3, BICR56 cell lines. Specificity of GLUT-1 pAb (clone A 3536) [9], TKTL1 mAb (clone JFC12T10) [49] and Ki-67 mAb (clone MIB-1) [50] have been previously demonstrated. Protein extraction from OSCC cell lines BICR3 and BICR56 was performed as described previously [51]. Normal human oral mucosal tissue protein was purchased from BioChain (Hayward, CA, USA) as control. The membranes were analyzed by immunoblotting using IGF-R1β, HK 2, PFK-1, LDHA, SDHA, SDHB, and ATP synthase antibodies (Additional file 2: Table S1), or IgG control antibodies (BD Pharmingen, Heidelberg), and monoclonal mouse anti-human GAPDH (Abcam, Cambridge, UK, dilution: 1:5000) specific primary antibody overnight at 4°C. Binding of the primary antibodies was detected with HRP-conjugated goat anti-mouse or goat anti-rabbit secondary antibody (Santa Cruz Biotechnology, CA, USA) and visualized by the enhanced chemiluminescence method (GE Healthcare, Freiburg, Germany).

Quantification of western blot bands was carried out by using an automated densitometric quantification digitizing system (UN-SCAN-IT Gel software, version 6.1, Silk Scientific, Inc., Utah, USA) [39].

### Real-time polymerase chain reaction (qPCR) analysis

To analyze gene expression of IGF-R1, GLUT-1, HK 2, PFK-1, TKTL1, SDHA, SDHB, and ATP synthase by RT-PCR, we extracted total cellular RNA and performed cDNA synthesis from OSCC cell lines (BICR3, BICR56) as previously described [52]. Gene expression of LDHA in OSCC cell lines has been shown previously [39]. The amount of total RNA was determined by measuring absorbance at 260 nm. The purity of the total RNA was established by confirming that the 260 nm: 280 nm ratio was within a 1.8-2.0 range, indicating that the RNA preparations were free of contaminants. Normal human oral keratinocyte cDNA (HOK cDNA) was purchased by ScienCell (Carlsbad, CA, USA) as control. The reference genes GAPDH and beta-actin were used for relative quantification and cDNA quality (integrity) control. To quantitate mRNA expression, qPCR with the LightCycler System (Roche Applied Science, Mannheim, Germany) was established as described before [53]. Commercial primer kits were purchased from Search LC (Heidelberg, Germany). Melt-curve analysis was be used to identify specific reaction products. The relative quantification value, fold difference, is expressed as 2^-ΔΔCt^ as described previously [54].

### Statistical analysis

Statistical analysis was performed with MedCalc Software, Version 13.1.1 (Mariakerke, Belgium). Data were analyzed using the non-parametric Mann–Whitney *U* Test or Kruskal-Wallis test when more than 2 groups were compared. Correlation analysis of TUNEL assay or Ki-67 with metabolism-related proteins was performed by the non-parametric Spearman Rho rank correlation coefficient. All p-values presented were 2-sided and p < 0.05 was considered statistically significant.

## Results

### Expression of IGF-R1β, glycolysis-related proteins GLUT-1, HK 2, PFK-1, LDHA, TKTL1, mitochondrial enzymes SDHA, SDHB, and ATP synthase in normal mucosa, oral precursor lesions and OSCC

**Figure 1:**
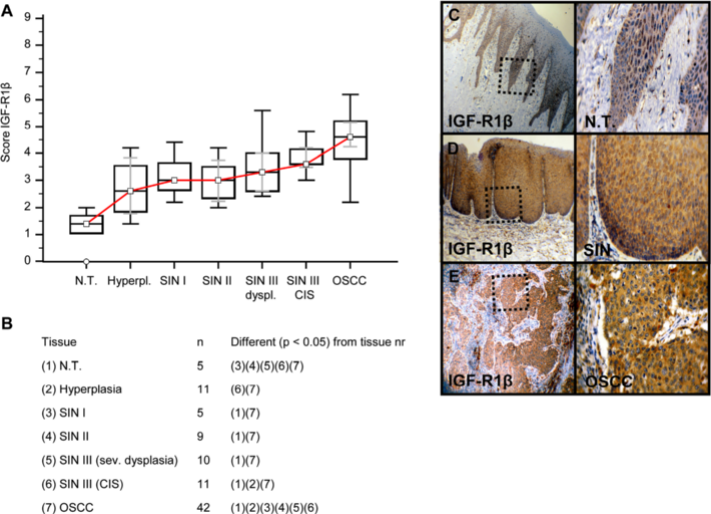
**Immunohistochemical analysis and staining of IGF-R1β in normal oral mucosal tissue, oral precursor lesions - hyperplasia, SIN I, SIN II, SIN III, and invasive OSCC.** In comparison to normal tissue/hyperplasia a significantly (p < 0.05, Kruskal-Wallis Test; **A** and **B**) increased expression of IGF-R1β is observed in OSCC. IGF-R1β expression is significantly increased in OSCC compared with SIN I-III (p < 0.0001, Mann–Whitney *U* Test). Analysis refers to averaged scores. Red line indicates IGF-R1β expression results during carcinogenesis. Grey lines show 95% confidence intervals. Analysis of significant statistically different single values is indicated in the table below **(B)**. SIN III is subdivided in severe dysplasia (sev. dysplasia) and carcinoma *in situ* (CIS). IGF-1R, insulin-like growth factor-1 receptor; SIN, squamous intraepithelial neoplasia; N.T., normal tissue. Immunohistochemical staining shows representative images of IGF-R1β expression in N.T. **(C)**, SIN **(D)**, and OSCC **(E)**. Brown chromogen color (3,3′-Diaminobenzidine) indicates positive staining, the blue color shows the nuclear counterstaining by hematoxylin. The square box demonstrates the area of interest (original magnification: x100-fold, left panel) which is also shown in larger magnification (x200-fold, right panel).

**Figure 2:**
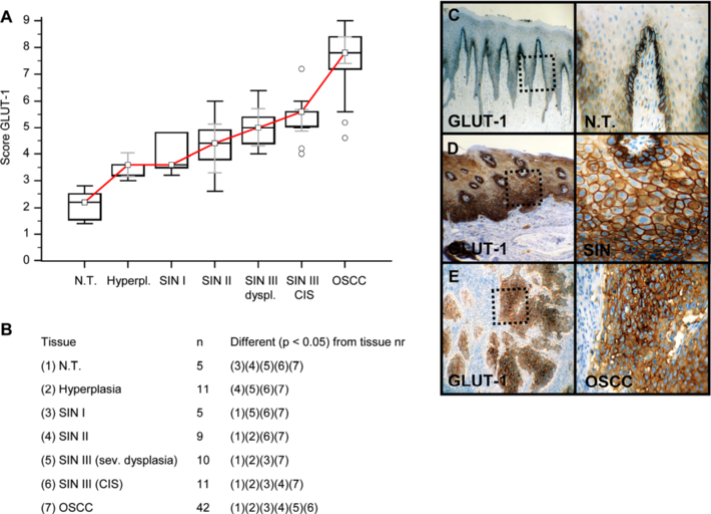
**Immunohistochemical analysis and staining of GLUT-1 in normal oral mucosal tissue, oral precursor lesions - hyperplasia, SIN I, SIN II, SIN III, and invasive OSCC.** In comparison to normal tissue/hyperplasia a significantly (p < 0.05, Kruskal-Wallis Test; **A** and **B**) increased expression of GLUT-1 is observed in OSCC. GLUT-1 expression is significantly increased in OSCC compared with SIN I-III (p < 0.0001, Mann–Whitney *U* Test). Analysis refers to averaged scores. Red line indicates GLUT-1 expression results during carcinogenesis. Grey lines show 95% confidence intervals. Analysis of significant statistically different single values is indicated in the table below **(B)**. SIN III is subdivided in severe dysplasia (sev. dysplasia) and carcinoma *in situ* (CIS). GLUT-1, glucose transporter-1; SIN, squamous intraepithelial neoplasia; N.T., normal tissue. Immunohistochemical staining shows representative images of GLUT-1 expression in N.T. **(C)**, SIN **(D)**, and OSCC **(E)**. Brown chromogen color (3,3′-Diaminobenzidine) indicates positive staining, the blue color shows the nuclear counterstaining by hematoxylin. The square box demonstrates the area of interest (original magnification: x100-fold, left panel) which is also shown in larger magnification (x200-fold, right panel).

**Figure 3:**
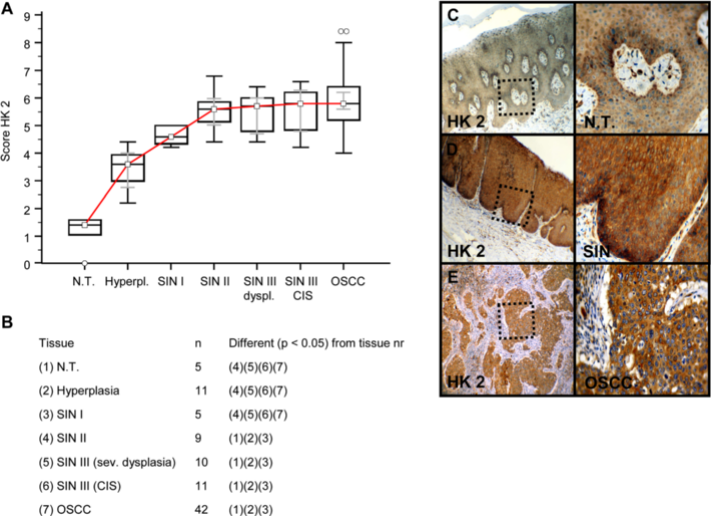
**Immunohistochemical analysis and staining of HK 2 in normal oral mucosal tissue, oral precursor lesions - hyperplasia, SIN I, SIN II, SIN III, and invasive OSCC.** In comparison to normal tissue/hyperplasia a significantly (p < 0.05, Kruskal-Wallis Test; **A** and **B**) increased expression of HK 2 is observed in OSCC. HK 2 expression is significantly increased in OSCC compared with SIN I-III (p < 0.0397, Mann–Whitney *U* Test). Analysis refers to averaged scores. Red line indicates HK 2 expression results during carcinogenesis. Grey lines show 95% confidence intervals. Analysis of significant statistically different single values is indicated in the table below **(B)**. SIN III is subdivided in severe dysplasia (sev. dysplasia) and carcinoma *in situ* (CIS). HK 2, hexokinase 2; SIN, squamous intraepithelial neoplasia; N.T., normal tissue. Immunohistochemical staining shows representative images of HK 2 expression in N.T. **(C)**, SIN **(D)**, and OSCC **(E)**. Brown chromogen color (3,3′-Diaminobenzidine) indicates positive staining, the blue color shows the nuclear counterstaining by hematoxylin. The square box demonstrates the area of interest (original magnification: x100-fold, left panel) which is also shown in larger magnification (x200-fold, right panel).

**Figure 4:**
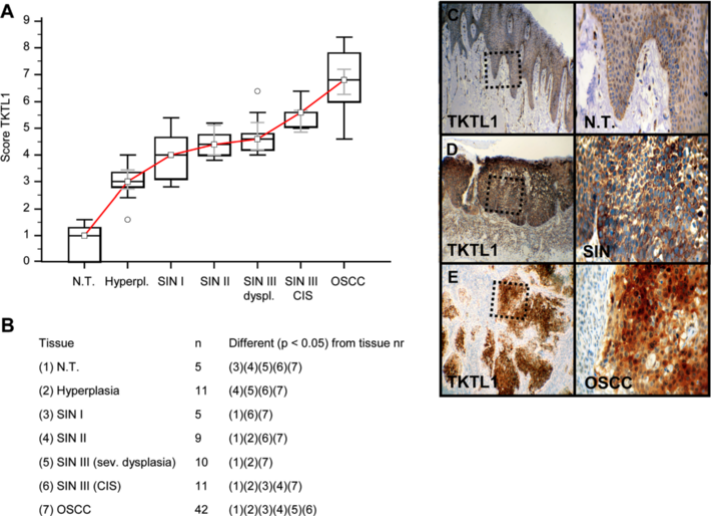
**Immunohistochemical analysis and staining of TKTL1 in normal oral mucosal tissue, oral precursor lesions - hyperplasia, SIN I, SIN II, SIN III, and invasive OSCC.** In comparison to normal tissue/hyperplasia a significantly (p < 0.05, Kruskal-Wallis Test; **A** and **B**) increased expression of TKTL1 is observed in OSCC. TKTL1 expression is significantly increased in OSCC compared with SIN I-III (p < 0.0001, Mann–Whitney *U* Test). Analysis refers to averaged scores. Red line indicates TKTL1 expression results during carcinogenesis. Grey lines show 95% confidence intervals. Analysis of significant statistically different single values is indicated in the table below **(B)**. SIN III is subdivided in severe dysplasia (sev. dysplasia) and carcinoma *in situ* (CIS). TKTL1, transketolase-like-1; SIN, squamous intraepithelial neoplasia; N.T., normal tissue. Immunohistochemical staining shows representative images of TKTL1 expression in N.T. **(C)**, SIN **(D)**, and OSCC **(E)**. Brown chromogen color (3,3′-Diaminobenzidine) indicates positive staining, the blue color shows the nuclear counterstaining by hematoxylin. The square box demonstrates the area of interest (original magnification: x100-fold, left panel) which is also shown in larger magnification (x200-fold, right panel).

**Figure 5:**
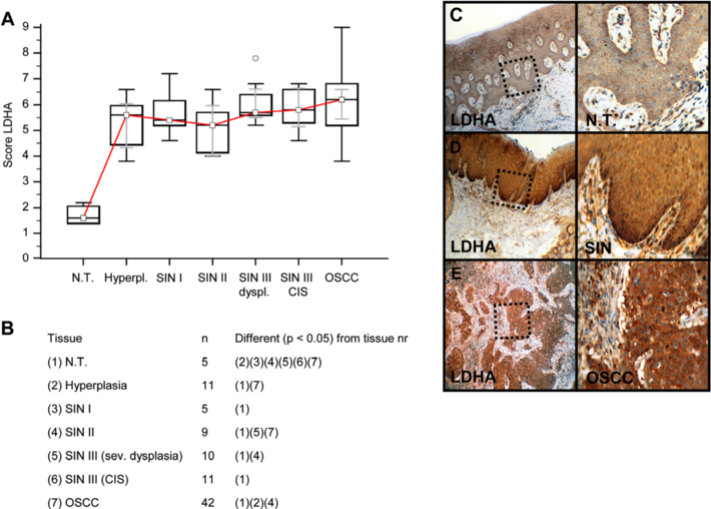
**Immunohistochemical analysis and staining of LDHA in normal oral mucosal tissue, oral precursor lesions - hyperplasia, SIN I, SIN II, SIN III, and invasive OSCC.** In comparison to normal tissue/hyperplasia a significantly (p < 0.05, Kruskal-Wallis Test; **A** and **B**) increased expression of LDHA is observed in OSCC. No difference of LDHA expression in OSCC is observed in comparison with SIN I-III (p = 0.0822, Mann–Whitney *U* Test). Analysis refers to averaged scores. Red line indicates LDHA expression results during carcinogenesis. Grey lines show 95% confidence intervals. Analysis of significant statistically different single values is indicated in the table below **(B)**. SIN III is subdivided in severe dysplasia (sev. dysplasia) and carcinoma *in situ* (CIS). LDHA, lactate dehydrogenase; SIN, squamous intraepithelial neoplasia; N.T., normal tissue. Immunohistochemical staining shows representative images of LDHA expression in N.T. **(C)**, SIN **(D)**, and OSCC **(E)**. Brown chromogen color (3,3′-Diaminobenzidine) indicates positive staining, the blue color shows the nuclear counterstaining by hematoxylin. The square box demonstrates the area of interest (original magnification: x100-fold, left panel) which is also shown in larger magnification (x200-fold, right panel).

**Figure 6:**
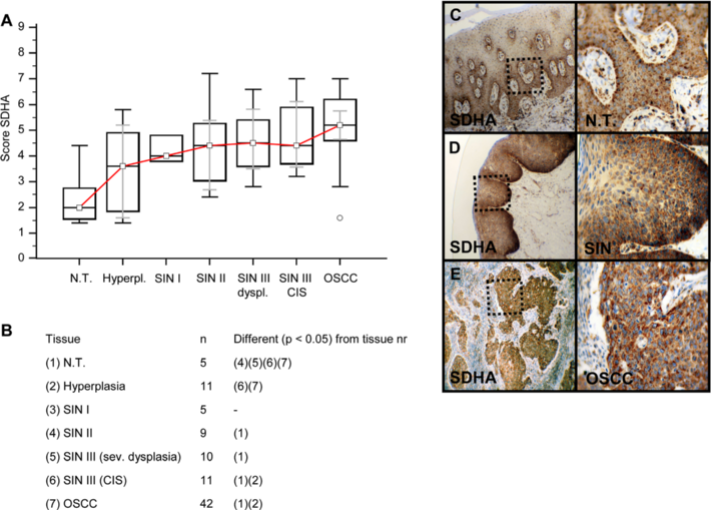
**Immunohistochemical analysis and staining of SDHA in normal oral mucosal tissue, oral precursor lesions - hyperplasia, SIN I, SIN II, SIN III, and invasive OSCC.** In comparison to normal tissue/hyperplasia a significantly (p < 0.05, Kruskal-Wallis Test; **A** and **B**) increased expression of SDHA is observed in OSCC. SDHA expression is significantly increased in OSCC compared with SIN I-III (p = 0.0103, Mann–Whitney *U* Test). Analysis refers to averaged scores. Red line indicates SDHA expression results during carcinogenesis. Grey lines show 95% confidence intervals. Analysis of significant statistically different single values is indicated in the table below **(B)**. SDHA is subdivided in severe dysplasia (sev. dysplasia) and carcinoma *in situ* (CIS). SDHA, succinate dehydrogenase A; SIN, squamous intraepithelial neoplasia; N.T., normal tissue. Immunohistochemical staining shows representative images of SDHA expression in N.T. **(C)**, SIN **(D)**, and OSCC **(E)**. Brown chromogen color (3,3′-Diaminobenzidine) indicates positive staining, the blue color shows the nuclear counterstaining by hematoxylin. The square box demonstrates the area of interest (original magnification: x100-fold, left panel) which is also shown in larger magnification (x200-fold, right panel).

**Figure 7:**
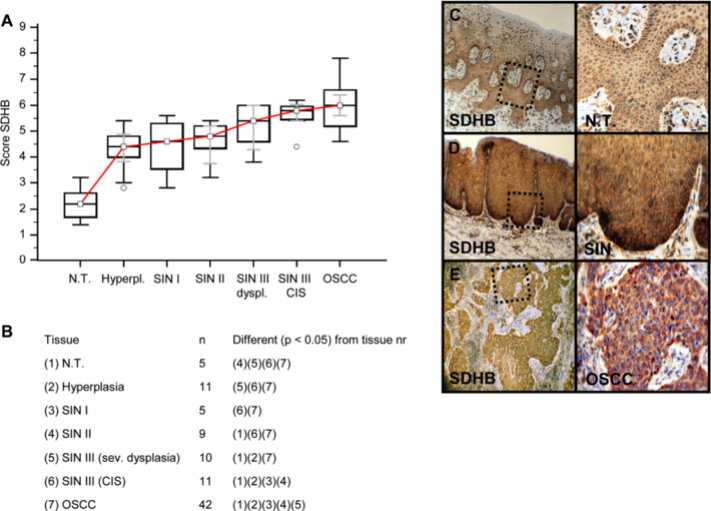
**Immunohistochemical analysis and staining of SDHB in normal oral mucosal tissue, oral precursor lesions - hyperplasia, SIN I, SIN II, SIN III, and invasive OSCC.** In comparison to normal tissue/hyperplasia a significantly (p < 0.05, Kruskal-Wallis Test; **A** and **B**) increased expression of SDHB is observed in OSCC. SDHB expression is significantly increased in OSCC compared with SIN I-III (p = 0.0001, Mann–Whitney *U* Test). Analysis refers to averaged scores. Red line indicates SDHB expression results during carcinogenesis. Grey lines show 95% confidence intervals. Analysis of significant statistically different single values is indicated in the table below **(B)**. SDHA is subdivided in severe dysplasia (sev. dysplasia) and carcinoma *in situ* (CIS). SDHB, succinate dehydrogenase B; SIN, squamous intraepithelial neoplasia; N.T., normal tissue. Immunohistochemical staining shows representative images of SDHB expression in N.T. **(C)**, SIN **(D)**, and OSCC **(E)**. Brown chromogen color (3,3′-Diaminobenzidine) indicates positive staining, the blue color shows the nuclear counterstaining by hematoxylin. The square box demonstrates the area of interest (original magnification: x100-fold, left panel) which is also shown in larger magnification (x200-fold, right panel).

**Figure 8:**
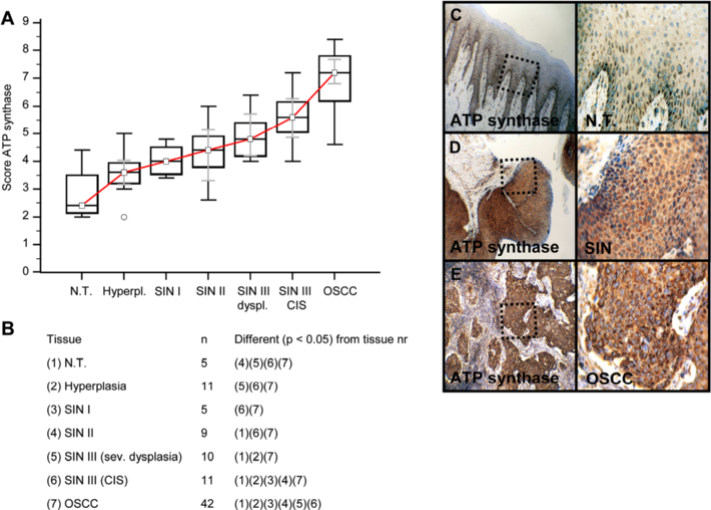
**Immunohistochemical analysis and staining of ATP synthase in normal oral mucosal tissue, oral precursor lesions - hyperplasia, SIN I, SIN II, SIN III, and invasive OSCC.** In comparison to normal tissue/hyperplasia a significantly (p < 0.05, Kruskal-Wallis Test; **A** and **B**) increased expression of ATP synthase is observed in OSCC. ATP synthase expression is significantly increased in OSCC compared with SIN I-III (p < 0.0001, Mann–Whitney *U* Test). Analysis refers to averaged scores. Red line indicates ATP synthase expression results during carcinogenesis. Grey lines show 95% confidence intervals. Analysis of significant statistically different single values is indicated in the table below **(B)**. ATP synthase is subdivided in severe dysplasia (sev. dysplasia) and carcinoma *in situ* (CIS). SIN, squamous intraepithelial neoplasia; N.T., normal tissue. Immunohistochemical staining shows representative images of ATP synthase expression in N.T. **(C)**, SIN **(D)**, and OSCC **(E)**. Brown chromogen color (3,3′-Diaminobenzidine) indicates positive staining, the blue color shows the nuclear counterstaining by hematoxylin. The square box demonstrates the area of interest (original magnification: x100-fold, left panel) which is also shown in larger magnification (x200-fold, right panel).

**Figure 9:**
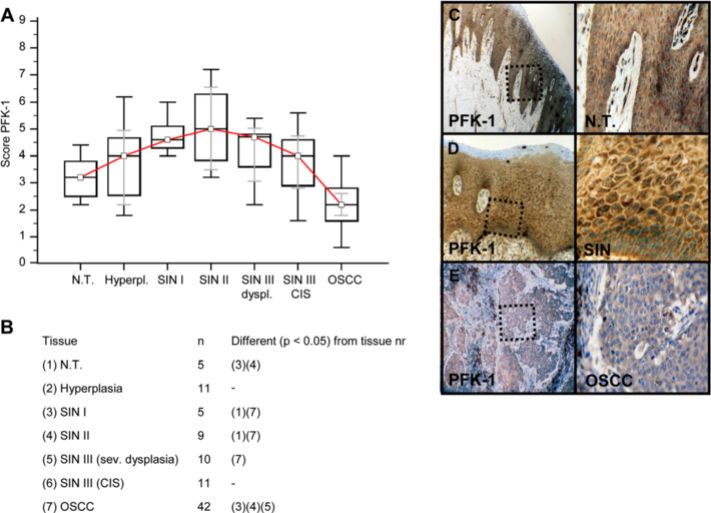
**Immunohistochemical analysis and staining of PFK-1 in normal oral mucosal tissue, oral precursor lesions - hyperplasia, SIN I, SIN II, SIN III, and invasive OSCC.** In comparison with normal tissue a significantly increased expression of PFK-1 expression is observed in SIN I and SIN II lesions (p < 0.05, Kruskal-Wallis Test; **A** and **B**). In comparison with SIN I, SIN II, and SIN III (sev. dysplasia) a significantly decreased expression of PFK-1 expression is observed in OSCC. PFK-1 expression is significantly decreased in OSCC compared with SIN I-III (p < 0.0001, Mann–Whitney *U* Test). Analysis refers to averaged scores. Red line indicates PFK-1 expression results during carcinogenesis. Grey lines show 95% confidence intervals. Analysis of significant statistically different single values is indicated in the table below **(B)**. SIN III is subdivided in severe dysplasia and carcinoma *in situ* (CIS). PFK-1, phosphofructokinase-1; SIN, squamous intraepithelial neoplasia; N.T., normal tissue. Immunohistochemical staining shows representative images of PFK-1 expression in N.T. **(C)**, SIN **(D)**, and OSCC **(E)**. Brown chromogen color (3,3′-Diaminobenzidine) indicates positive staining, the blue color shows the nuclear counterstaining by hematoxylin. The square box demonstrates the area of interest (original magnification: x100-fold, left panel) which is also shown in larger magnification (x200-fold, right panel).

Invasive OSCC of immunohistochemical stained serial sections was confirmed by H&E staining (Additional file 1: Figure S1). In comparison to normal tissue and hyperplasia a significantly (p < 0.05) increased expression of IGF-R1β (Figure 1), GLUT-1 (Figure 2), HK 2 (Figure 3), TKTL1 (Figure 4), LDHA (Figure 5), SDHA (Figure 6), SDHB (Figure 7), and ATP synthase (Figure 8) was observed in cancer cells of OSCC. Compared with SIN I-III PFK-1 expression (Figure 9) was significantly decreased in OSCC.

### Correlation of proliferation activity (Ki-67) with metabolic markers (GLUT-1, HK 2, PFK-1, LDHA, TKTL1, SDHA, SDHB, and ATP synthase) in OSCC serial sections

For investigation of proliferating cancer cells and its relation to metabolic characteristics, we performed correlation analysis of Ki-67 with GLUT-1, HK 2, LDHA, TKTL1, SDHA, SDHB, and ATP synthase in OSCC. Evaluation of immunohistochemically stained FFPE slides were measured by observer related semi-quantitative scoring and showed a strong positive correlation of Ki-67+ expression with metabolic active cancer cells as observed in OSCC serial sections. Significant correlation of proliferating cancer cells was observed with GLUT-1, TKTL1 mitochondrial markers SDHA, SDHB, and ATP synthase: GLUT-1 (rho = 0.370, 95% CI = 0.0750 to 0.606, p = 0.0157), TKTL1 (rho = 0.460, 95% CI = 0.165 to 0.704, p = 0.0056), SDHA (rho = 0.485, 95% CI = 0.213 to 0.688, p = 0.0011), SDHB (rho = 0.657, 95% CI = 0.441 to 0.801, p < 0.001), and ATP synthase (rho = 0.413, 95% CI = 0.125 to 0.637, p = 0.0065). No significant correlation of proliferation activity was found with HK 2 (rho = 0.152, 95% CI = -0.159 to 0.436, p = 0.3365) and LDHA (rho = 0.153, 95% CI = -0.158 to 0.437, p = 0.3336). Moreover, a significant correlation of GLUT-1 with TKTL1 in OSCC was analyzed (rho = 0.419, 95% CI 0.131 to 0.641, p = 0.0058) as previously indicated by our work. There was a significant inverse correlation of PFK-1 with TKTL1 detected (rho = -0.475, 95% CI -0.619 to -0.301, p < 0.0001).

### Correlation of apoptotic activity (TUNEL assay) with metabolic markers (GLUT-1, HK 2, PFK-1, LDHA, TKTL1, SDHA, SDHB, and ATP synthase) in OSCC serial sections

**Figure 10:**
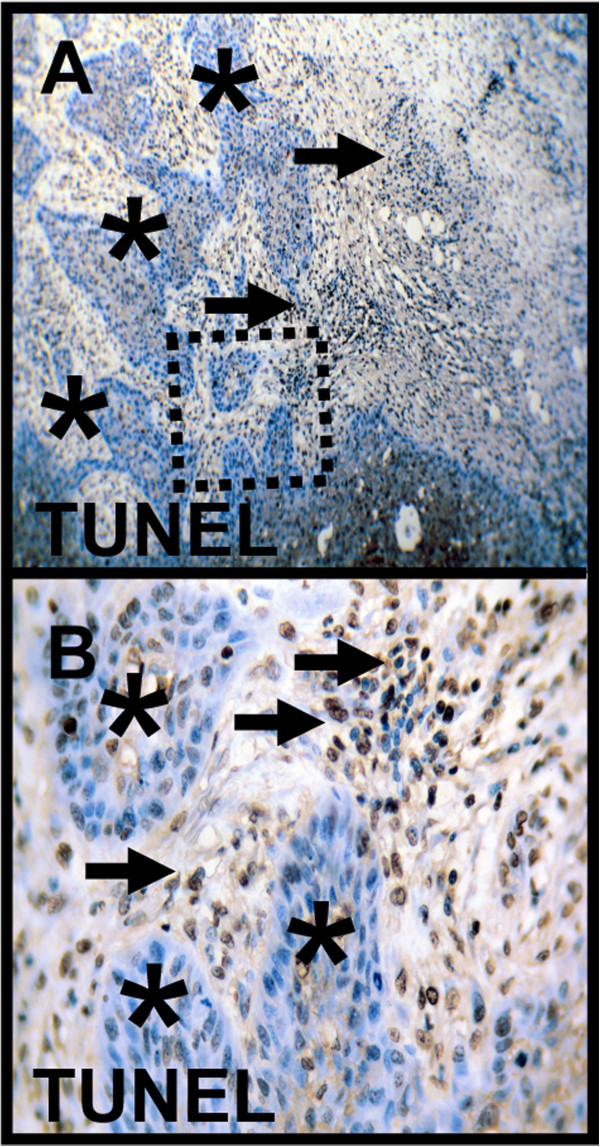
***In situ *detection of DNA fragmentation by TUNEL-staining in OSCC.** In metabolic highly active regions as demonstrated by markers IGF-R1β, HK 2, TKTL1, PFK-1, LDHA, SDHA, SDHB, and ATP synthase cancer cells do not undergo apoptosis (serial section, asterisk, **A**). In contrast, tumor-infiltrating leucocytes adjacent to the tumor demonstrate increased apoptotic activity (arrow, **B**). Brown chromogen color (3,3′-Diaminobenzidine) indicates positive staining, the blue color shows the nuclear counterstaining by hematoxylin. Original magnification: x200-fold. TUNEL, terminaldeoxynucleotidyl transferase-mediated deoxyuridinetriphosphate nick-end labeling.

For the investigation of apoptotic properties in cancer cells caused by OXPHOS (due to putative increased free radical production) we performed TUNEL assays in OSCC. Although tumor cells show increased OXPHOS-related enzymes (SDHA, SDHB, ATP-synthase, Figures 6, 7 and 8) no apoptotic activity (AI < 10%) in cancer cells was observed in those highly metabolic active regions (Figure 10). In contrast, tumor-infiltrating leucocytes adjacent to the tumor demonstrated increased apoptotic activity (Figure 10).

### IGF-R1β, HK 2, PFK-1, LDHA, SDHA, SDHB, ATP synthase antibody specifity is confirmed by western blot analysis

**Figure 11:**
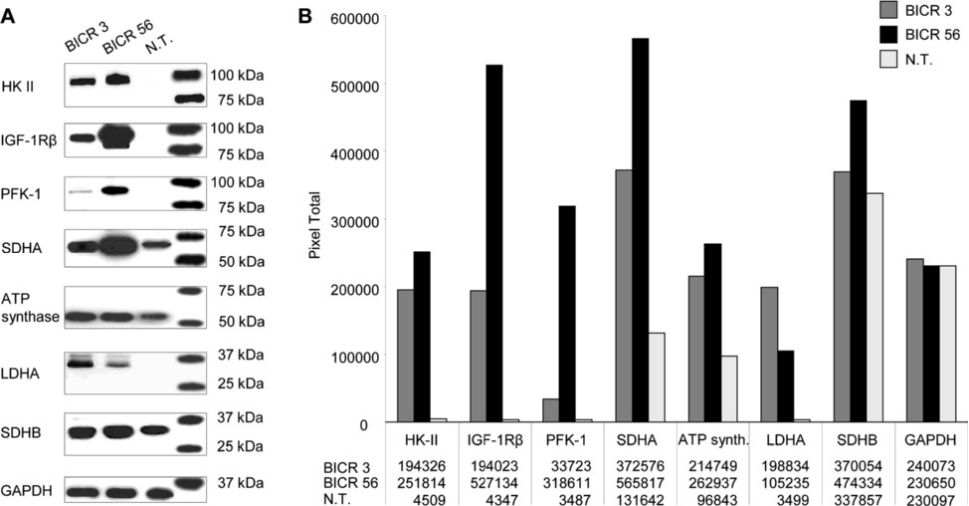
**Western blot analysis in normal tissue, BICR3 and BICR56 OSCC cell lines.** Western Blot of HK 2, IGF-R1β, PFK-1, SDHA, ATP synthase, LDHA, and SDHB in BICR3 and BICR56 OSCC cell lines confirm immunohistochemical staining specifity of antibodies (left panel, **A**). Western blot analysis shows increased HK 2 (~102 kDa), IGF-R1β (~97 kDa), PFK-1 (~85 kDa), SDHA (~70 kDa), ATP synthase (~53 kDa), LDHA (~37 kDa), and SDHB (~32 kDa) expression compared to normal tissue. GAPDH (loading control) is shown as a band of approximately 35 kDa. Densitometric quantification **(B)** of western blot protein bands (pixel total) is given in the right panel **(B)**. IGF-1R, insulin-like growth factor-1 receptor; HK 2, hexokinase 2; PFK-1, phosphofructokinase-1; LDHA, lactate dehydrogenase A; SDH, succinate dehydrogenase; GAPDH, glycerinaldehyd-3-phosphat-dehydrogenase; N.T., normal tissue.

Western Blot analysis of HK 2, IGF-R1β, PFK-1, SDHA, ATP synthase, LDHA, and SDHB in BICR3 and BICR56 OSCC cell lines confirmed immunohistochemical staining specifity of antibodies used in immunohistochemistry (Figure 11).

### Analysis of IGF-R1, GLUT-1, HK 2, PFK-1, TKTL1, SDHA, SDHB, and ATP synthase gene expression

IGF-R1, GLUT-1, HK 2, PFK-1, TKTL1, SDHA, SDHB, and ATP synthase gene expression in OSCC cell lines was increased in comparison to normal human oral keratinocytes (Table 1).

**Table 1:** Increased gene expression analysis (qPCR) of cancer metabolism-related proteins in OSCC cell lines compared with normal human oral keratinocytes (x-fold difference)

	**IGF-1R**	**GLUT-1**	**HK 2**	**PFK-1**	**TKTL1**	**SDHA**	**SDHB**	**ATP synthase**
BICR3	12.6-fold	22.4-fold	13.6-fold	2.8-fold	3.3-fold	14.7-fold	7.7-fold	10.3-fold
BICR56	18.2-fold	25.3-fold	11.9-fold	8.4-fold	4.9-fold	16.0-fold	6.6-fold	14.1-fold

## Discussion

In our study, we investigated cancer metabolism-related proteins in the carcinogenesis of OSCC. For the first time, we found increased expression of mitochondrial enzymes (SDHA, SDHB, ATP synthase) in OSCC compared with normal oral mucosa. However, very few data is available describing a mitochondrial oxidative metabolism [27] in OSCC. Authors assume that OXPHOS is an important pathway for the generation of ATP [11,22,23] and ROS [18,55-58] during the carcinogenesis of OSCC. The TUNEL assay demonstrated that tumor cells do not undergo apoptosis and therefore, increased ROS generation by OXPHOS does not reach toxic levels. Based on our results and as currently stated by Whitaker-Menezes *et al.*[57] in the context of breast cancer we assume that mitochondria are the ‘Achilles heel’ and ‘powerhouse’ in the carcinogenesis of OSCC [23,56-59]. Increased levels of ROS in tumor cells are generated by altered metabolic activity, oncogene activation, and deregulated proliferation [60]. Oncogenic transformation promotes the production of excessive ROS, which would become toxic if not counteracted, while low levels of ROS can help to promote cell proliferation. This is the reason why many cancer cells may show an increased expression of antioxidant proteins [26] such as LDHA [39] and TKTL1 [7] as indicated by our observation, which contribute to the survival and success of the tumor. Indeed, this dependence on antioxidants can make cancer cells more vulnerable to the inhibition of these detoxifying systems than normal cells, which do not harbor such a high burden of oxidative stress [61-63]. On the other hand, an increase in ATP production by OXPHOS has been shown in response to hypoxic stress and protects cells from a critical energy crisis [64]. However, we do not know which metabolic pathway (glycolysis vs. OXPHOS) has been upregulated in carcinogenesis of OSCC as first.

In the literature, bioactive food components [5,17,65,66] have been demonstrated to mediate the reversal of a glycolytic phenotype in cancer cells, thus leading to growth inhibition and induction of apoptosis (Table 2). The reprogramming of energy metabolism [67-70] has been suggested for targeting of mitochondria [18,19,21,23,55,58,61-63,71-74] and subsequent induction of apoptosis [71] as a valid anti-cancer strategy [18] for which bioactive food components [19] have been suggested. Rapidly proliferating cells are more sensitive to radio-, and chemotherapy, which have been shown to be less effective in non-dividing cancer cells [75]. Activation of mitochondrial OXPHOS [58,61] and other mechanisms in cancer cells by natural compounds may induce apoptosis even in therapy resistant cancer cells [55]. Because OXPHOS is the predominant supplier of ATP in (proliferating and) non-proliferating cancer cells [21] targeted anti-mitochondrial therapies could be of interest for apoptosis induction in quiescent (non-proliferating) but metabolically active cancer cells, which rely on mitochondrial lipid β-oxidation [76]. Therefore, bioactive food components inducing apoptosis by ROS generation (Table 2) and other mechanisms play an emerging role in cancer therapy. According to other tumor entities several other natural compounds have been shown to activate ROS [58,61] in OSCC [77-81] and subsequent apoptosis in cancer cells and may therefore provide a clear rational to study them in further pre-clinical and clinical trials (Table 2). Moreover, phytochemicals [82] and vitamins have different hypoxia-inducible factor-1 (HIF-1) binding capacities (inhibitory activity: lycopene > curcumin > tocopherol > ascorbic acid) suggestive for their impact on the decrease in tumor hypoxia and antioxidative properties in normal tissue [83].

**Table 2:** Bioactive food components (natural or synthetic compounds/vitamin derivatives) targeting mitochondria (ROS generation) and/or glycolysis that may act as sensitizer for chemoprevention and (neo-)adjuvant therapies in cancer treatment

			**Tumor entity**		***In-vitro/in-vivo *** **mechanism on tumor cells (OSCC/HNSCC/other tumor entities)**			**Pre-clinical *(in-vitro/in-vivo) *** **chemoprevention in cancer development**		**Clinical data**	
Compounds (polyphenols*, isothiocyanates, terpen/carotinoid** vitamins, derivates, fatty acids)			OSCC [66,84]/HNSCC	other	Apoptosis↑ (ROS↑ [18,23], Caspasen↑)	Glykolysis↓ [17] (mTOR↓ [31,85,86], HIF-1↓ [83], enzymes↓)	PPP↓ [87] (*e.g.* TKTL1↓)			Successful [88],*** approach in prospective clinical trials	
	natural	synthetic						OSCC [66,84]/HNSCC	other	OSCC [66,84]/HNSCC	Other
Curcumin* (turmeric) [83,85,88-102]	X	-	Yes [92-95,101]	Yes	Yes [96] (ROS↑) [93]	Yes (mTOR↓ [85,94,102]; HIF-1↓ [83,100])	n.d.	Yes [92-94,101]	Yes	Yes (phase I) [95,97]	Yes (phase II)
Resveratrol* (grapes) [85,103-114]	X	-	Yes [104]	Yes	Yes, (ROS↑ [107])	Yes [109] (mTOR↓ [85,103,110,111]; HIF-1↓ [109])	n.d.	Yes [107,112]	Yes	n.d.	Yes
EGCG* (green tea) [66,84,85,88,104,115-119]	X	-	Yes [66,104,117]	Yes	Yes, (ROS↑ [117])	Yes (mTOR↓ [85]; HIF-1↓ [118,119])	n.d.	Yes [66,104,116]	Yes	Yes (phase II) [66,84,115]	Yes (phase II)
Ellagic acid*, (**Pro-**) Anthocyanins* (berrys) [66,84,120-127]	X	-	Yes [66,84]	Yes	Yes, (ROS↑ [122-124])	Yes (mTOR↓ [124,125]; HIF-1↓ [126])	n.d.	Yes [66,84,120,121,127]	Yes	Yes (phase II) [66,84]	Yes
Genistein* (soyabeans) [85,88,128-133]	X	-	Yes [128,129]	Yes	Yes, (ROS↑ [130])	Yes (mTOR↓ [85,131]; HIF-1↓ [132,133])	n.d.	Yes [128,129]	Yes	n.d.	Yes (phase II)
Apigenin* (parsley) [134-141]	X	-	Yes [134-137]	Yes	Yes, (ROS↑ [135])	Yes (mTOR↓ [138]; HIF-1↓ [139,140])	n.d.	Yes [134-136]	Yes	n.d.	Yes
								No [137]			
Quercetin* (fruits/vegetables) [141-150]	X	-	Yes [142-144,149,150]	Yes	Yes, (ROS↑ [144,145])	Yes (mTOR↓ [146,147]; HIF-1↓ [147,148])	n.d.	Yes [142-144,149]	Yes	Yes [150]	Yes
ITC, glucosinolates (cruciferous vegetables) [85,151-157]	X	-	Yes [152-154,157]	Yes	Yes, (ROS↑ [155])	Yes (mTOR↓ [85,151]; HIF-1↓ [157])	n.d.	Yes [152-154,157]	Yes	n.d.	Yes (phase I)
Lycopene** (tomato) [83,158-169]	X		Yes [158,163,164]	Yes	Yes (ROS↑↓) [160-162]	Yes (mTOR↓ [167-169]; HIF-1↓ [83])	n.d.	Yes [158,163,164]	Yes [163]	n.d. [163]	Yes (phase II)
Vit. A** (retinoids) [101,170-175]	X	-	Yes [101,170,171,174,175]	Yes	Yes, (ROS↑ [172])	Yes (mTOR↓ [173]; HIF-1 n.d.)	n.d.	Yes [101]	Yes	Yes [174,175]; No (phase III) [170,171]	Yes
Vit. D [176-181]	X	X [179]	Yes [177,178]	Yes	Yes, (ROS↑ [181])	Yes (mTOR↓ [179]; HIF-1↓ [180])	n.d.	Yes [177,178]	Yes	n.d.	Yes
Vit. E (γ-T3) [83,176,182-185]	X	-	n.d.	Yes	Yes, (ROS↑ [183])	Yes (mTOR↓ [184]; HIF-1↓ [182,185])	n.d.	n.d.	Yes	n.d.	n.d.
Vit. C + K [83,186-194]	X	X	Yes [186]	Yes	Yes (ROS↑ [191])	Yes (mTOR↓ [192]; HIF-1↓ [83,187,193])	n.d.	Yes [186]	Yes	n.d.	Yes (Phase I/II)
Oxybenfotiamine [195]	-	X	n.d.	Yes	Yes	n.d.	Yes	n.d.	n.d.	n.d.	n.d.
Benzoquinone (wheat germ extract) [196-198]	X	-	Yes [197]	Yes	Yes (Caspasen↑ [197], ROS n.d.)	Yes [198] (mTOR n.d.; HIF-1 n.d.)	Yes [197,198]	n.d.	Yes	Yes (Phase II/III) [197]	Yes (Phase II/III)
PUFAs (n-3/n-6 family) [199-203]	X	-	Yes [200,201]	Yes	Yes (ROS↑ [202])	Yes [199] (mTOR↓ [202]; HIF-1↓ [199,203])	n.d.	Yes [200]	Yes	Yes (Phase II) [201]	Yes (Phase II)

Most compounds may decrease glycolytic activity by targeting mTOR/HIF-1 pathway and increase apoptotic activity by ROS generation in cancer cells. Focused on OSCC most experience is available for polyphenols (flavonoids: EGCG, anthocyanins, in bold).

ROS, reactive oxygen species; Caspasen, cysteinyl-aspartate specific protease; OSCC, oral squamous cell carcinoma; HNSCC, head and neck squamous cell carcinoma; HIF, Hypoxia-inducible factor; mTOR, mammalian target of rapamycin; EGCG, epigallocatechin-3-gallate; ITC, isothiocyanate; γ-T3, gamma-tocotrienol; Vit., vitamin; PUFAs, polyunsaturated fatty acids; n.d., no data; ***chemopreventive outcome: decrease of precursor lesions or decrease of cancer biomarkers or decrease of secondary malignancies or increase in patient survival or increase in quality of life or reduction of toxic side effects of radio- and/or chemotherapy. The arrow indicates an increase (↑) or decrease (↓) in levels, phosphorylation status or activity of the different signals.

Lactate, pyruvate, gluthathione, and NADPH generated in glycolysis and/or the PPP effectively scavenge free radicals and ROS, thereby protecting the tumor cell from free radical-mediated DNA damage [26] (*e.g.* radiation therapy) or other ROS-inducing therapies by natural compounds leading to apoptosis. Most likely, modulation of one pathway will be not effective in most cases [17]. Therefore, synchronous [59] targeting of glycolysis (*e.g.* carbohydrate-restricted diets [16,204-217] or natural compounds, Table 2) with anti-mitochondrial therapies [18,19,21,23,55,58,61-63,71-74] increasing ROS (Table 2) may act as sensitizer for adjuvant therapies in OSCC or could be useful for chemoprevention. Based on the literature a synergistic effect of a carbohydrate-restricted diet with an anti-mitochondrial therapy can be concluded, since carbohydrate-restricted diets may induce enhanced OXPHOS and lead to inhibition of mTOR [218], which is responsible for synthesis of glycolytic enzymes [30,31]. Specifically observed in patients with head and neck cancer a ketogenic diet decreased the *in vivo* production of lactate in tumor cells [213].

However, it must be stated that natural compounds like phytochemicals [65,219-225] and vitamins may also prevent ROS-mediated carcinogenicity in cancer chemoprevention. During carcinogenesis ROS may act as a double-edged sword [226]. ROS are important intermediates of cellular signaling that suppress and promote tumorigenesis at once. They make both mitochondrial DNA and nuclear DNA susceptible to damage, and mutations in these two DNA pools are reported to lead to carcinogenesis [227]. However, targeted anti-mitochondrial therapies inducing apoptosis probably require functional active mitochondria without mutations that may respond to radiotherapy/chemo-radiotherapy in OSCC [228].

With specific regard to SDHA and SDHB, vitamin E (α-tocopheryl succinat, target: respiratory complex II in mitochondria) [229] and resveratrol (target: respiratory complex V in mitochondria, ATP synthase) [230] were shown to induce apoptosis in cancer cells. Metformin has been demonstrated to block respiratory complex I in mitochondria [231] as an effective anti-cancer agent [232] and prevented the development of OSCC from carcinogen-induced premalignant lesions [233]. More recently, a synthetic modified thiamine analog oxybenfotiamine [195] specifically inhibits TKTL1 in the PPP [87], of which elevated levels have been detected in the carcinogenesis of OSCC [7]. Targeting the PPP [87] as a detoxifying system [26] may revise tumor hypoxia and resistance to radio- and chemotherapy [7,9]. Therefore, small molecules like oxybenfotiamine [195] provide new opportunities for targeted therapies in cancer and specifically OSCC. Nevertheless, the cytoprotective function of the PPP is not limited to defending against ROS but also expands to helping DNA damage repair [70].

However, it remains unclear whether phytochemicals are standardized effective for chemoprevention [2,17,65,66,84,88,115,120,219,221,223,234,235] in the treatment of precursor lesions or OSCC development as suggested for multistep carcinogenesis [2] but they provide a clear rational for further *in-vitro, in-vivo*, and clinical studies in the carcinogenesis of OSCC (Table 2) [2,84,88,115,120,219,234-236]. Polyphenols like flavonoids and anthocyanidins have been well investigated in pre-clinical and clinical trials for the treatment of oral precursor lesions and OSCC [84,115,234]. For example, in 1999 Li *et al.* have already been reported of the chemopreventive impact of green tea on oral leukoplakia with increased rate of partial regression (systemically, oral capsules with 1.2 g polyphenols, and topical tea extract in glycerine over a period of 6 months) [236].

Proliferating cells have intrinsic increased metabolic activities compared to non-proliferating cells [21,69]. This is supported by our data showing a significantly correlation of proliferating cancer cells with both glycolysis-related proteins (GLUT-1, TKTL1), and OXPHOS-related enzymes (SDHA, SDHB, ATP synthase). In this context glycolysis-related proteins may act as detoxifying system [26] (LDHA, TKTL1) of increased ATP producing (and ROS generating) OXPHOS-related proliferating cancer cells. These findings can be clinically addressed by differentiating cancer patients into metabolic responders and non-responders for malignancies such as SCC of the esophagus or head and neck squamous cell carcinoma [237-239].

As for OSCC, there are several reports for glycolysis [9] as the predominant energy metabolism pathway. Glycolysis is involved in aggressive tumor behavior because it causes radio-, and chemotherapy resistance, creates a tumor microenvironment favorable for tumor cell migration, induces angiogenesis, and contributes to the immunologic escape of tumors [26]. However, a previous study by Yi *et al.* demonstrated that inhibition of the glycolysis-related PFK-1 activity redirects the glucose flux through the PPP [240], thereby conferring a selective growth advantage on cancer cells. Our results are well in line with this hypothesis showing increased TKTL1 expression and decreased PFK-1 expression in OSCC (significant inverse correlation). Zhang *et al.*[8] presented a similar mechanism describing a metabolic shift from glycolysis into the PPP [67] in OSCC. The authors conclude that the highly robust nature of OSCC metabolism implies that a systematic medical approach targeting multiple metabolic pathways is needed to improve cancer treatment. Downregulation of PFK-1 as observed in our study can be explained by an increase of natural inhibitors such as ATP, which is generated by OXPHOS, and citrate (from the citric acid cycle) that inhibits PFK-1 expression [241]. Therefore, we assume a metabolic shift [8,67,240,241] of glucose from glycolysis towards the PPP mediated by the increased presence of PFK-1 inhibitors like ATP/citrate generated in OXPHOS (indicated by SDHA, SDHB, ATP synthase expression) during the carcinogenesis of OSCC.

If not provided by glycolysis, metabolites (pyruvate) for lactate production are available from amino acids [242]. Amino acid catabolism from the citric acid cycle (*e.g.* glutaminolysis) supports pyruvate anabolism leading to lactate and NADPH production [69,242]. NADPH, pyruvate, and lactate itself have been proven to scavenge free radicals, thus protecting cancer cells from apoptosis [26]. However, this hypothesis of lactate anabolism through amino acids catabolism requires further studies in OSCC. Glutamine metabolism is also a cancer cell metabolic pathway important for both ATP production and providing intermediates for macromolecular synthesis. However, Glucose, not glutamine, was described as the dominant energy source required for proliferation and survival of head and neck squamous carcinoma cells [243]. This result does not automatically exclude lactate generation by amino acid catabolism, as the glutamine pathway has not been described for OSCC and has yet to be revealed. Finally, focusing on combination strategies [116,158,186,244] (Table 2) with different signaling pathways (*e.g.* mTOR) [245] that have the potential to eradicate malignant and premalignant clones are warranted [245,246].

For the first time, our study provides evidence of increased IGF-1R in OSCC. The expression of IGF-1R has been described for *in-vitro* analysis of an OSCC cell line [247] but not for carcinogenesis of OSCC yet. The authors state that IGF-1R activation is associated with resistance of EGFR-tyrosine-kinase inhibitor (TKI) treatment. Therefore, targeting IGF-1R pathway, reversal of hyperinsulinemia and IGF by dietry recommendations [16,34,199,204-217,248] or metformin [232] may decrease resistance of EGFR-TKI as well as reduce the risk of cancer recurrence in tumor patients [34].

## Conclusions

This study provides the first evidence of the expression of glycolysis-related proteins GLUT-1, HK 2, PFK-1, LDHA, TKTL1 and mitochondrial enzymes SDHA, SDHB, ATP synthase in the multi-step carcinogenesis of OSCC. It seems that both, hypoxia-related glucose metabolism and mitochondrial oxidative phosphorylation characteristics are associated with the carcinogenesis of OSCC. Acidosis and OXPHOS may drive a metabolic shift towards the PPP [67]. Therefore, inhibition of the PPP and glycolysis, as well as targeted anti-mitochondrial therapies (ROS generation) by natural compounds (polyphenol mix, selective vitamins) or synthetic vitamin derivatives (*e.g.* oxybenfotiamine) may act as sensitizer for apoptosis in cancer cells mediated by adjuvant therapies in OSCC. Summarizing in other words, targeting detoxifying systems (*e.g.* TKTL1, LDHA) make cancer cells or (oral) precursor lesions more vulnerable to apoptosis.

## Abbreviations

ATP: Adenosine Triphosphate; GLUT-1: glucose transporter-1; HK 2: Hexokinase 2; HIF-1: Hypoxia-inducible factor-1; IGF-1R: Insulin-like growth factor-I receptor; IGF: Insulin-like growth factor; LDHA: Lactate dehydrogenase A; mTOR: Mammalian target of rapamycin; OSCC: Oral squamous cell carcinoma; OXPHOS: (Mitochondrial) Oxidative phosphorylation; PFK-1: Phosphofructokinase-1; PPP: Pentose phosphate pathway; RTK: Receptor tyrosine kinase; SDH: Succinate dehydrogenase; SIN: Squamous intraepithelial neoplasia; TKI: Tyrosine-kinase inhibitor; TKTL1: Transketolase-like-1.

## Competing interests

The authors have no competing interests.

## Authors’ contributions

MG and SR conceived the study, performed the coordination and drafted the manuscript. MC, ML and AM carried out immunohistochemistry studies, cell culture, and western blot analysis. TB analysed histopathological specimen and carried out immunohistochemistry studies. PT and MG performed qPCR analysis. PT and WK carried out the data collection and performed the statistical analyses. All authors read and approved the final manuscript.

## Supplementary Material

Additional file 1: Figure S1 Hematoxylin-Eosin (H&E) staining. H&E staining shows representative images of normal tissue **(A)**, squamous intraepithelial neoplasia SIN I **(B)**, SIN II **(C)**, SIN III carcinoma *in situ***(D)** and invasive OSCC **(E)**. Original magnification: x100-fold. N.T., normal tissue. Click here for file

Additional file 2: Table S1 Clonality, host species, dilution, and company of antibodies used for immunohistochemistry and western blot analysis [249-252]. Click here for file
