# Supplementary figures and images for: Association of cancer metabolism-related proteins with oral carcinogenesis – indications for chemoprevention and metabolic sensitizing of oral squamous cell carcinoma?
Source: J Transl Med. 2014 Jul 21;12:208. doi: 10.1186/1479-5876-12-208 (PMC4110933; doi:10.1186/1479-5876-12-208)

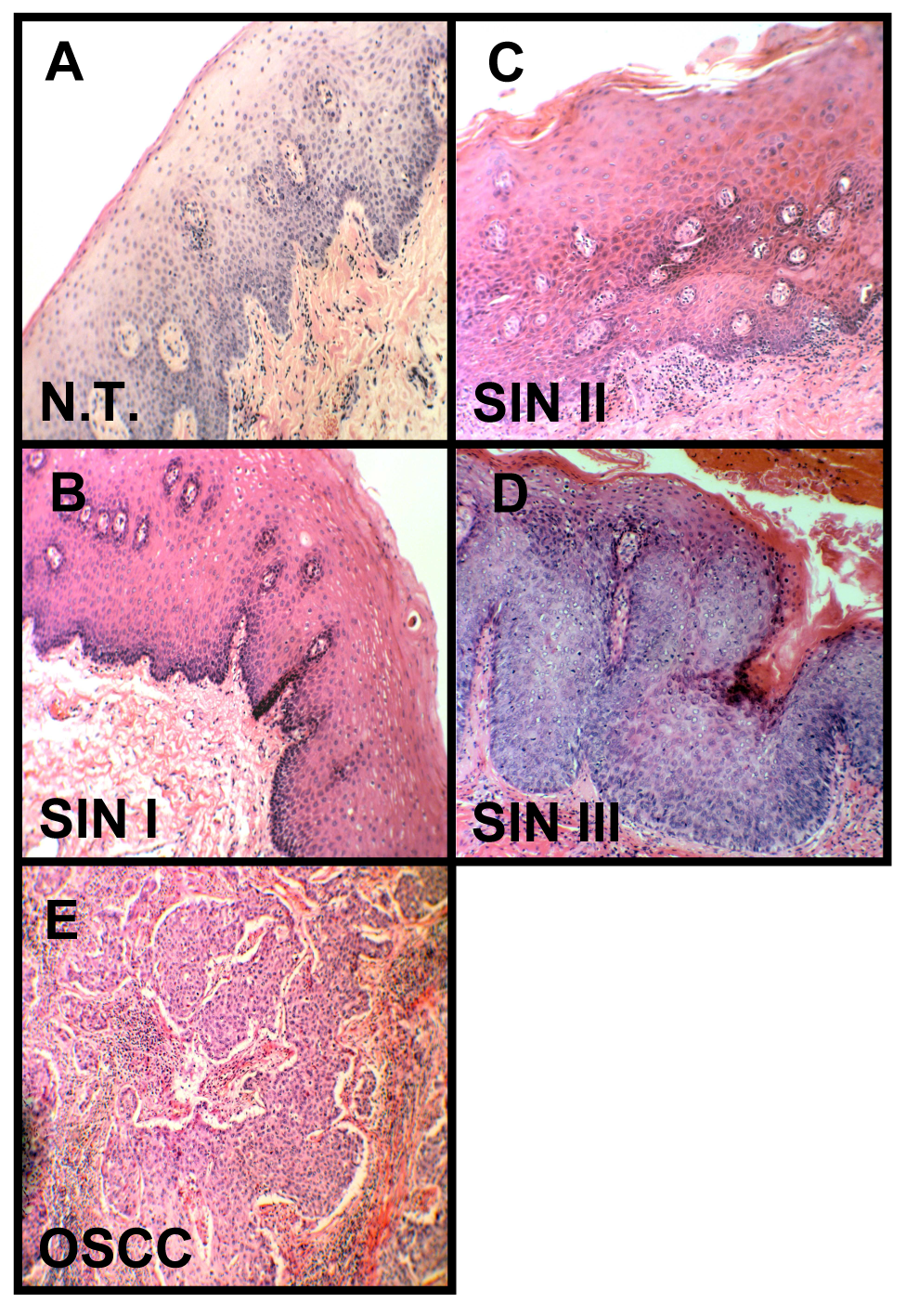

Supplement: Additional file 1: Figure S1 — Hematoxylin-Eosin (H&E) staining. H&E staining shows representative images of normal tissue (A), squamous intraepithelial neoplasia SIN I (B), SIN II (C), SIN III carcinoma in situ(D) and invasive OSCC (E). Original magnification: x100-fold. N.T., normal tissue. [file 1479-5876-12-208-S1.tiff]
