# Supplementary material for: Association of cancer metabolism-related proteins with oral carcinogenesis – indications for chemoprevention and metabolic sensitizing of oral squamous cell carcinoma?
Source: J Transl Med. 2014 Jul 21;12:208. doi: 10.1186/1479-5876-12-208 (PMC4110933; doi:10.1186/1479-5876-12-208)
Supplement: Additional file 2: Table S1 — Clonality, host species, dilution, and company of antibodies used for immunohistochemistry and western blot analysis [249-252]. [file 1479-5876-12-208-S2.doc]

**Additional file 2: Table S1: Clonality, host species, dilution, and company of antibodies used for immunohistochemistry and western blot analysis.**

| Antibody | Host species/ clonality | Dilution | | Company | Function |
| --- | --- | --- | --- | --- | --- |
|  |  | IHC | WB |  |  |
| IGF-1Rβ | Rabbit / polyclonal | 1:50 | 1:500 | Santa Cruz, CA, USA | RTK that stimulates growth and survival of cancer cells, blocks apoptosis; the β subunit contains a tyrosine kinase enzymatic domain; ligands: IGFs, Insulin; target of new antineoplastic pharmacologic agents . |
| GLUT-1 | Rabbit / polyclonal | 1:200 | - | Dako, Hamburg, Germany | Glucose transporter-1 is the main transporter involved in glucose influx . |
| HK 2 | Mouse / monoclonal | 1:70 | 1:500 | Santa Cruz, CA, USA | Key enzyme of the glycolysis; HK 2 is involved in increased utilization of glucose by cancer cells; target of new antineoplastic pharmacologic agents . |
| PFK-1 | Rabbit / polyclonal | 1:200 | 1:500 | Novus Biologicals, Cambridge, UK | Key enzyme of the glycolysis; PFK-1 is inhibited by ATP and citrate (from the citric acid cycle) . |
| LDHA  (=LDH5) | Rabbit / monoclonal | 1:400 | 1:1000 | Cell Signaling, Frankfurt, Germany | Executes the final step of aerobic glycolysis, favors the conversion of pyruvate to lactate; target of new antineoplastic pharmacologic agents . |
| TKTL1 | Mouse / monoclonal | 1:200 | - | TAVARTIS, Hainburg, Germany | Key enzyme of the modified PPP; generation of Acetyl-CoA (lipid synthesis) and nucleic acids; target of new antineoplastic pharmacologic agents . |
| SDHA  (=Flavoprotein) | Rabbit / monoclonal | 1:200 | 1:1000 | Cell Signaling, Frankfurt, Germany | SDH is the only enzyme that participates in both the citric acid cycle and the electron transport chain . It´s a key component and oxidates succinate to fumarate with the [reduction](http://en.wikipedia.org/wiki/Redox) of [ubiquinone](http://en.wikipedia.org/wiki/Ubiquinone) to [ubiquinol](http://en.wikipedia.org/wiki/Ubiquinol) during the citric acid cycle as a component of respiratory complex II in mitochondria. |
| SDHB  (=Iron-sulfurprotein) | Rabbit / monoclonal | 1:200 | 1:50000 | Abcam, Cambridge, UK | SDH is the only enzyme that participates in both the citric acid cycle and the electron transport chain . It´s a key component and oxidates succinate to fumarate with the [reduction](http://en.wikipedia.org/wiki/Redox) of [ubiquinone](http://en.wikipedia.org/wiki/Ubiquinone) to [ubiquinol](http://en.wikipedia.org/wiki/Ubiquinol) during the citric acid cycle as a component of respiratory complex II in mitochondria. |
| ATP synthase | Mouse / monoclonal | 1:500 | 1:1000 | Abcam, Cambridge, UK | Synthesizes ATP from ADP as a component of the respiratory complex V in mitochondria . |
| Ki-67 | Mouse / monoclonal | 1:200 | - | Dako, Hamburg, Germany | Nuclear antigen associated with cell proliferation and found throughout the cell cycle (G1 S, G2, M-phases), and absent in resting cells (G0 phase) . |
| IHC, Immunohistochemistry; WB, Western Blot; IGF-1R, Insulin-like growth factor-1 receptor; RTK, Receptor Tyrosine Kinase; IGF, Insulin-like growth factor; GLUT-1, glucose transporter-1; HK 2, Hexokinase 2; PFK-1, Phosphofructokinase-1; ATP, Adenosine Triphosphate; LDHA, Lactate dehydrogenase A; TKTL1, Transketolase-like-1; PPP, pentose phosphate pathway; SDH, Succinate dehydrogenase. | | | | | |
